# Supplementary material for: New failure mechanism for evaluating ultimate inclined load adjacent to slope
Source: PLoS One. 2023 Jul 27;18(7):e0289015. doi: 10.1371/journal.pone.0289015 (PMC10374118; doi:10.1371/journal.pone.0289015)
Supplement: S1 Appendix — (DOCX) [file pone.0289015.s001.docx]

**Appendix A**

The equations are supposed:

*$\beta_{i}=\frac{1}{2}\cot\phi\ln\frac{\sigma_{i}}{\sigma_{0}}+\theta_{i}$* (1a)

*$\alpha_{i}=\frac{1}{2}\cot\phi\ln\frac{\sigma_{i}}{\sigma_{0}}-\theta_{i}$* (1b)

where *α_i_* and *β_i_* are the *i*th *α* and *β* slip line families, *σ_i_* and *θ_i_* are the characteristic stress and angle of the *i*th slip line family, *σ*_0_ is constant.

According to the characteristics of *β* slip line family at slope crest:

*$\beta_{i}=\beta_{1}=\frac{1}{2}\cot\phi\ln\frac{\sigma_{1}}{\sigma_{0}}+\theta_{1}$* (2)

According to (1a) - (1a) and (1a) + (1a):

*$\beta_{i}=2\theta_{i}+\alpha_{i}$* (3a)

*$\sigma_{i}=\sigma_{0}e^{(\beta_{i}+\alpha_{i})\tan\phi}$* (3b)

By substituting equation (2) into equation (3a):

*$\alpha_{i}=\frac{1}{2}\cot\phi\ln\frac{\sigma_{1}}{\sigma_{0}}+\theta_{1}-2\theta_{i}$* (4)

By substituting equation (2) and (4) into equation (3b):

*$\sigma_{2}=\sigma_{i}=\sigma_{1}e^{2(\theta_{1}-\theta_{i})\tan\phi}$* (5)

where$\theta_{2}=\theta_{i}=\theta_{1}+k\frac{\Delta\theta}{N}$, *k*=0~*N*,$\Delta\theta=\theta_{3}-\theta_{1}$, and *N* is the point partition of Riemann boundary.
