# Supplementary material for: New failure mechanism for evaluating ultimate inclined load adjacent to slope
Source: PLoS One. 2023 Jul 27;18(7):e0289015. doi: 10.1371/journal.pone.0289015 (PMC10374118; doi:10.1371/journal.pone.0289015)
Supplement: Attachment — Submitted filename: Response to Reviewers-YX-5 Jun.docx [file pone.0289015.s002.docx]

Author's Reply to the Review Report

We would like to thank the Reviewers for their comments and constructive suggestions which have greatly helped improve the paper. We have considered these comments and revised the manuscript accordingly. All changes in the revision are indicated in blue.

**Reviewer #1**

(1) The paper proposes a new failure mechanism for calculating the ultimate inclined load adjacent to the slope. The topic is interesting, and the model could be useful for engineering practice. However, the paper lacks geotechnical discussion in general. Furthermore, the reasons for the selected range of geotechnical properties are not given.
**Answer:**

We have revised the paper such that more geotechnical discussion or applications are laid out. See new Figs. 6 (b) and 7(e). For calculation the ultimate inclined load adjacent to slope, the geotechnical properties include the unit weight (γ), cohesion (*c*) and internal friction angle (*φ*), footing width (B), slope height (H), slope angle (*η*), the inclination angle (*δ*). This paper has comprehensively considered the above geotechnical properties, e.g., H/B varying from 1 to 2.5 in lines 177, 191; *c/γ*B varying from 0.5 to 0.25 in lines 177, 239, and section 4.3; *φ* varying from 20^0^ to 40^0^ , see line 209 in section 4.1; *η* varying from 15^0^ to 60^0^, see line 239 in section 4.2; *δ* varying from 0^0^ to 40^0^ in Figs. 6, 7 and 8. According to the results of this study with the above geotechnical properties, the selected range of geotechnical properties are very general and can be applicable to a wide range of engineering practice.

(2) Besides, the model proposes the evaluation of convex critical slip surfaces. However, the critical slip surfaces presented in Figs. 6 and 9 are concave.

**Answer:**

Note that the critical slope contour (i.e., the line OD in Fig.1) is not the critical slip surface, see lines 165-167. Slip line field theory considered that the critical slope contour is concave [14-17]. The convex critical slope contour OD is found in this study. Section 2.2.3 and Figure 4 give the formation mechanism of the critical slope contour changing from a concave to a convex as the load becomes larger. The proposed method is to calculate the ultimate inclined load first and then determine the critical slip surface (i.e., the failure model). The calculation flow chart is shown in Figure 3(b).

Figure 5 (a) gives the calculation diagram of the ultimate load as the critical slope contour changes from concave to convex. Figure 5 (b) gives the critical slip surface (concave) of the proposed method using the shear strain rate. Therefore, the critical slip surfaces in Figs. 6 and 9 are not the critical slope contour. Figure 5 (c), (d), Figure 6 (c), (d), (e), (f), and Figure 9 all indicate the critical slip surface are concave using SLIDE (Bishop method) and FLAC (Finite difference method). Therefore, the critical slip surface (concave) is not contradictory to the critical slope contour from concave to convex curve proposed in this paper.

(3)The manuscript and methods are interesting. However, the quality must be improved to be considered for publication. The mechanisms involved in slope stability and the choices made for modelling have to be better explained.

**Answer:**

The legend of the expression of the proposed failure mechanism, i.e., three states (i.e., unstable, limiting equilibrium, stable states) corresponding to the different positions of the critical slope contour, is added in Figure 3(a). The expression statement of the proposed failure mechanism is modified in the manuscript, see line 165, 303-304. The selection judgment step in Fig. 3(b) is modified to make it clear that the load imposed at the slope top surface is the ultimate inclined load when the critical slope contour is tangent to the slope surface.

(4) Also, it is crucial to clearly state the limitations of the proposed model. Moreover, the numerical model is not compared to experimental data, so the accuracy of the results could not be assessed.

**Answer:**

The limitations of the proposed method is added in line 318, i.e., the proposed method needs further study for the non-homogeneous case. The feasibility of the proposed method is verified by the definition of the ultimate load, i.e., FSs calculated using SLIED5.0 (Bishop method) and FLAC7.0 (Finite difference method) are close to 1.0 when the ultimate inclined load is imposed at the slope top surface, see Figure 6 (c), (e), Figure 9 (b), (d), and Table 1.

In the Revised Manuscript, the UB and LB-FELA solution calculated using OptumG2 (see line 213) are added in new Figures 6 (b), 7 (e).

In new Figure 6 (b), *N*_u_ values calculated by the proposed method are close to those obtained from UB and LB-FELA using OptumG2. However, *N*_u_ values calculated by the current method of characteristics (CMOC) and FELA in [10] are close to those of the UB-FELA using OptumG2 when *φ* is 40^0^.

Fig.7(e) shows that *N*_u_ values calculated by CMOC are much larger than the values of UB and LB-FELA using OptumG2. However, *N*_u_ values calculated by the proposed method are close to the mean values of the UB and LB-FELA using OptumG2.

The larger the strength of the slope is, the higher the ultimate inclined load is. Thus, *N*_u_ increases with *c/γ*B increasing. Fig.8 shows that CMOC gave an unreasonable result because *N*_u_ decreases as *c/γ*B increases from 0.25 to 0.5. *N*_u_ values calculated by the proposed method are close to the mean values of the UB and LB-FELA using OptumG2 with *c/γ*B = 0.25, 0.5.

Comparing with the existing methods, e.g., SLIED5.0 (Bishop method), FLAC7.0 (Finite difference method) and the UB and LB-FELA using OptumG2, the proposed method can get the reasonable ultimate inclined load. The model experiments will be studied in the future.

**Reviewer #2**

(1) Please provide an additional explation for the Riemann boundary, state the factorized conditions in thetab, the differecne between the Riemann and mixed boundary condition must be clearly defined, whcih factors must be considered in order to decrease the inlination or failure
**Answer:**

Equation 33 has a small error, which was corrected, see line 140. To make the difference between the Riemann and mixed boundary condition clearer, a new equation 36 has been added, see line 148.

(2) please add the standard limitation for the FS value, compared the methods which shows lower FS and how the current proposed model could be classified as good over the other methods
**Answer:**

According to the definition of the ultimate load, i.e., FS is close to 1.0 when the ultimate inclined load is imposed at the slope top surface. As shown in Figure 6 (c), (e), Figure 9 (b), (d), and Table 1, FSs of the failure model calculated by the proposed method are close to 1.0. However, FSs calculated by CMOC are smaller than 1.0 as shown in Figure 6 (d), (f), and Figure 9 (c), (e). Thus, the ultimate inclined loads calculated by the proposed method are more reasonable, and it is better than CMOC.

(3) add a brief recommendation section

**Answer:**

The recommendation section is added in lines 319-323.

**Journal Requirements:**

When submitting your revision, we need you to address these additional requirements.

1. Please ensure that your manuscript meets PLOS ONE's style requirements, including those for file naming. The PLOS ONE style templates can be found at

https://journals.plos.org/plosone/s/file?id=wjVg/PLOSOne_formatting_sample_main_body.pdf and

<https://journals.plos.org/plosone/s/file?id=ba62/PLOSOne_formatting_sample_title_authors_affiliations.pdf>

**Answer:**

The format has been revised as required, see line 4.

2. Thank you for stating the following financial disclosure:

“The research was supported by the science and technology research project of the education department of Jilin province. (No.JJKH20220280KJ).”

Please state what role the funders took in the study.  If the funders had no role, please state: "The funders had no role in study design, data collection and analysis, decision to publish, or preparation of the manuscript."

If this statement is not correct you must amend it as needed.

Please include this amended Role of Funder statement in your cover letter; we will change the online submission form on your behalf.

**Answer:**

The role of the fund is described in the text, see line 326.

3. In your Data Availability statement, you have not specified where the minimal data set underlying the results described in your manuscript can be found. PLOS defines a study's minimal data set as the underlying data used to reach the conclusions drawn in the manuscript and any additional data required to replicate the reported study findings in their entirety. All PLOS journals require that the minimal data set be made fully available. For more information about our data policy, please see http://journals.plos.org/plosone/s/data-availability.

Upon re-submitting your revised manuscript, please upload your study’s minimal underlying data set as either Supporting Information files or to a stable, public repository and include the relevant URLs, DOIs, or accession numbers within your revised cover letter. For a list of acceptable repositories, please see <http://journals.plos.org/plosone/s/data-availability#> loc-recommended-repositories. Any potentially identifying patient information must be fully anonymized.

Important: If there are ethical or legal restrictions to sharing your data publicly, please explain these restrictions in detail. Please see our guidelines for more information on what we consider unacceptable restrictions to publicly sharing data: http://journals.plos.org/plosone/s/data-availability#loc-unacceptable-data-access-restrictions. Note that it is not acceptable for the authors to be the sole named individuals responsible for ensuring data access.

We will update your Data Availability statement to reflect the information you provide in your cover letter.

**Answer:**

All the data used in the study are generated by using Matlab by inputing various variables. We have included a matlab file in the Revised manuscript and we believe all readers can replicate our results by using the program by inputing the relevant variables we have used in the manuscript. See Revised Cover letter.

4. We note that you have stated that you will provide repository information for your data at acceptance. Should your manuscript be accepted for publication, we will hold it until you provide the relevant accession numbers or DOIs necessary to access your data. If you wish to make changes to your Data Availability statement, please describe these changes in your cover letter and we will update your Data Availability statement to reflect the information you provide.

**Answer:**

All the data used in the study are generated by using Matlab by inputing various variables. We have included a matlab file in the Revised manuscript and we believe all readers can replicate our results by using the program by inputing the relevant variables we have used in the manuscript. See lines 389-708 in the Revised manuscript.

5. Please include a separate caption for each figure in your manuscript.

**Answer:**

The right diagram is a enlarged diagram from the left diagram in Figure 5(a). The word “Enlarge” was added in Revised manuscript in Figure 5(a). Figure 7(a) is replaced by four figures, i.e., Figure 7(a), (b), (c), (d).

6. Please upload a copy of Figures 11 and 14, to which you refer in your text on page 13 and 15. If the figure is no longer to be included as part of the submission please remove all reference to it within the text.

**Answer:**

The copies of Figures 11 and 14 in my text on page 13 and 15 are as follow. The reference is remove.

The copy of Figure 11(b) in [10], i.e., the values of CMOC in Figure 7(e) in Revised manuscript

The copy of Figure 14(c) in [10], i.e., Face bearing capacity failure in Figure 9(a) in Revised manuscript
